# Targeted tumor therapy: turning the genome on its head

**DOI:** 10.1186/2051-1426-1-S1-P133

**Published:** 2013-11-07

**Authors:** Matthew M Lawler, Ian S Dunn, Estelle E Newton, Lenora B Rose, James T Kurnick

**Affiliations:** 1 TriBiotica LLC, Madison, WI, USA

## 

TriBiotica has combined molecular biology of cancer and proprietary chemistry to implement an innovative therapeutic approach to cancer treatment. Instead of developing immunological reagents (such as cells, antibodies or chimeric receptors) targeting antigens already present on tumor cells, the new technology produces novel structures that are not native to the tumor cell. The generation of novel molecules in a tumor-specific manner accordingly allows implementation of immunotherapeutic interventions that recognize and destroy tumor cells. Unlike immunotherapy protocols that are limited by availability of target antigens, TriBiotica’s technology utilizes the tumor’s unique transcriptome to force tumor cells to produce novel molecules that are either toxic by themselves, or allow immunotherapeutic destruction of the tumor cells without inducing toxicity against normal cells. As a proof of concept, we have used an HPV16 template from a cervical carcinoma cell to create a FLAG-tag that is uniquely assembled in HPV16 expressing tumor cells. Of the cervical cancer cell lines Caski, HeLa, and C33A, only Caski contains the HPV16 E6/E7 oncogene. Each of these lines was treated with templated assembly of bio-orthogonal compounds designed to produce FLAG tag peptide specifically in the presence of HPV16 E6/E7 mRNA. At 24 hours post-treatment, in situ production of FLAG tag in each tumor type was assayed by ELISA. The results demonstrate that FLAG tag is selectively produced in Caski cells harboring the target HPV16 template. To our knowledge, this represents the first oncogene-templated synthesis of a peptide tag within living cells. The approach offers the potential for producing a wide variety of biologically-active molecules specifically in targeted cells, leaving normal cells unaffected. This technology offers a platform that links next-generation sequencing technologies to identify genetic markers of cancers as potential targets in a vast array of tumors. Combined with genetic analyses, the therapy is adaptable to the ever-changing genetic landscape of tumors, as the same novel effector molecule can be assembled on an almost limitless array of target nucleic acid templates, so that it is possible to adapt the formation of the novel therapeutic product molecule to any target genetic templates arising from the outgrowth subsets of tumor cells. Combined with appropriate diagnostic testing, the TriBiotica technology has the potential to create a new paradigm for targeting of tumor cells that opens the door to the treatment of virtually any tumor type. The technology relies on identifying specific tumor genetic templates, and utilizing a proprietary technology that assembles novel epitopes present only in the tumor cells.

